# Population Distribution of Beta-Lactamase Conferring Resistance to Third-Generation Cephalosporins in Human Clinical Enterobacteriaceae in The Netherlands

**DOI:** 10.1371/journal.pone.0052102

**Published:** 2012-12-20

**Authors:** Guido M. Voets, Tamara N. Platteel, Ad C. Fluit, Jelle Scharringa, Claudia M. Schapendonk, James Cohen Stuart, Marc J. M. Bonten, Maurine A. L. Hall, National ESBL Surveillance Working Group

**Affiliations:** 1 Department of Medical Microbiology, University Medical Centre Utrecht, Utrecht, The Netherlands; 2 SALTRO, Department of Medical Microbiology, Utrecht, The Netherlands; 3 National Institute for Public Health and the Environment, Bilthoven, The Netherlands; Institut National de la Recherche Agronomique, France

## Abstract

There is a global increase in infections caused by Enterobacteriaceae with plasmid-borne β-lactamases that confer resistance to third-generation cephalosporins. The epidemiology of these bacteria is not well understood, and was, therefore, investigated in a selection of 636 clinical Enterobacteriaceae with a minimal inhibitory concentration >1 mg/L for ceftazidime/ceftriaxone from a national survey (75% *E. coli*, 11% *E. cloacae*, 11% *K. pneumoniae*, 2% *K. oxytoca*, 2% *P. mirabilis*). Isolates were investigated for extended-spectrum β-lactamases (ESBLs) and *ampC* genes using microarray, PCR, gene sequencing and molecular straintyping (Diversilab and multi-locus sequence typing (MLST)). ESBL genes were demonstrated in 512 isolates (81%); of which 446 (87%) belonged to the CTX-M family. Among 314 randomly selected and sequenced isolates, *bla*_CTX-M-15_ was most prevalent (n = 124, 39%), followed by *bla*_CTX-M-1_ (n = 47, 15%), *bla*_CTX-M-14_ (n = 15, 5%), *bla*_SHV-12_ (n = 24, 8%) and *bla*_TEM-52_ (n = 13, 4%). Among 181 isolates with MIC ≥16 mg/L for cefoxitin plasmid encoded AmpCs were detected in 32 and 27 were of the CMY-2 group. Among 102 *E. coli* isolates with MIC ≥16 mg/L for cefoxitin *ampC* promoter mutations were identified in 29 (28%). Based on Diversilab genotyping of 608 isolates (similarity cut-off >98%) discriminatory indices of bacteria with ESBL and/or *ampC* genes were 0.994, 0.985 and 0.994 for *E. coli, K. pneumoniae* and *E.* cloacae, respectively. Based on similarity cut-off >95% two large clusters of *E. coli* were apparent (of 43 and 30 isolates) and 21 of 21 that were typed by belonged to ST131 of which 13 contained *bla*_CTX-M-15_. Our findings demonstrate that *bla*_CTX-M-15_ is the most prevalent ESBL and we report a larger than previously reported prevalence of *ampC* genes among Enterobacteriaceae responsible for resistance to third-generation cephalosporins.

## Introduction

The increasing prevalence of plasmid-borne β-lactamases in Enterobacteriaceae that confer resistance to third-generation cephalosporins is a world-wide problem. The most prevalent amongst these acquired β-lactamases are the Ambler class A ESBLs of the CTX-M, TEM and SHV families. [1] These ESBLs are capable of hydrolyzing penicillins, cephalosporins (except cephamycins), and monobactams and are inhibited by clavulanic acid. [2] An emerging class of β-lactamases are the plasmid-borne Ambler class C cephalosporinases (pAmpCs). [3] AmpC enzymes are capable of hydrolyzing penicillins, cephalosporins (although fourth-generation cephalosporins only weakly), and monobactams and are not inhibited by clavulanic acid. [3] The molecular epidemiology of these resistance mechanisms is largely unknown, as most large-scale molecular surveys were limited, either to certain species (e.g., *Escherichia coli* or *Klebsiella pneumoniae*), a specific environment (either hospital or general practice) or specimen type (e.g. urine or faeces). [4]–[9].

In the Netherlands the proportions of urine samples and blood cultures with *E. coli* (intermediate) resistant to third-generation cephalosporins increased from 2.6% and 2.6%, respectively in 2008 to 3.4% and 4.7%, respectively, in 2010 [10].

The aim of this study was to determine the population distribution of beta-lactamase conferring resistance to third-generation cephalosporins in an unbiased, cross-sectional, large and nation-wide sample of clinical isolates in the Netherlands.

## Materials and Methods

### Isolates

From February 1, 2009 until May 1, 2009, 31 Dutch microbiology laboratories were asked to submit all isolates of *E. coli*, *K. pneumoniae, Klebsiella oxytoca*, *Proteus mirabilis* and *Enterobacter cloacae* with a positive ESBL screen test (minimal inhibitory concentration (MIC)>1 mg/L for cefotaxime or ceftazidime or an ESBL alarm from the Phoenix or Vitek-2 expert system). The need for written consent of patients was waived by the ethical committee because of the retrospective nature of the study, the use of fully anonimized patient data only and because of the absence of any study related procedures. From each laboratory the first 25 consecutive isolates, if available, were included in this study, allowing for only 1 isolate per patient.

In a central laboratory screen tests were repeated using broth microdilution (BMD) (Merlin Diagnostic GmbH, Rüsselsheim, Germany) and only isolates with a confirmed positive test were included in this study. Susceptibility testing was performed for amikacin, cefotaxime +/− clavulanic acid, ceftazidime +/− clavulanic acid, chloramphenicol, ciprofloxacin, fosfomycin, gentamicin, nitrofurantoin, piperacillin/tazobactam, tobramycin and trimethoprim/sulfamethoxazole using Sensititre microbroth dilution plates (TREK Diagnostic Systems, East-Grinstead, England). MICs were interpreted according to EUCAST criteria.

For each isolate the following epidemiological data were collected: age (0–19, 20–59 and ≥60 years) and gender of the patient, specimen type (urine, faeces, wounds/skin, respiratory tract, blood and other (e.g., ascites, gynecological cultures)) and institution (hospital (university, non-university), general practitioner (GP), or long term care facility (LTCF)). The participating laboratories are geographically dispersed over the Netherlands and represent a mixture of secondary and tertiary care hospitals, LTCFs and GPs. The 31 laboratories serve 58 hospitals, covering approximately 45% of all hospital beds in the Netherlands.

### Molecular Characterization of Beta-lactamase Genes

The presence of ESBL genes was determined by Check-KPC ESBL microarray analysis (Cat. No. 10-0018, CheckPoints, Wageningen, The Netherlands), which detects single nucleotide polymorphisms (SNPs) and reports the presence of TEM or SHV SNPs associated with an ESBL phenotype and specifies CTX-M groups (CTX-M group 1, 2, 9, or combined 8/25) [1]. As the assay cannot provide a type number for TEM, SHV and CTX-M genes (http://www.lahey.org/Studies/), PCR and gene sequencing was performed for definite determination of ESBL genes as previously described [11]–[13]. From all screen-positive isolates a random sample of 314 isolates was taken for sequence-based confirmation of resistance genes. Isolates with a negative array result were first investigated using TEM, SHV, and CTX-M group-specific PCRs and, if negative, with multiplex PCRs for detecting other Ambler A class ESBL families (GES, PER and VEB) [14]. All PCR products were sequenced. In isolates with an AmpC phenotype (cefoxitin MIC ≥16 mg/L) the presence of pAmpC was determined by PCR and sequencing [14]. For *Enterobacter* spp., PCR results for plasmid ACT-1 and MIR-1,-2,-3 were not included because the primers used for these PCRs are based on primers that may also detect the chromosomal *ampC* of *Enterobacter* spp. [15]. If negative and no other β-lactamase was detected, the promoter of the chromosomal *ampC* of *E. coli* was sequenced to identify mutations associated with derepression [16]–[18]. For this PCR the following primers were designed: ECC-GS-F: GATCGTTCTGCCGCGTG and ECC-GS-R: GGGCAGCAAATGTGGAGCAA.

### Isolate Typing

*E. coli, K. pneumoniae, K. oxytoca* and *Enterobacter* spp. isolates were typed using DiversiLab (bioMérieux, Marcy l’Etoile, France) [19]. Representative *E. coli* isolates from dominant patterns identified by Diversilab were also analyzed by multi-locus sequence typing (MLST) (http://mlst.ucc.ie/mlst/dbs/Ecoli). Discriminatory index calculations were preformed using Ridom EpiCompare as previously described. [19].

### Statistical Analysis

Statistical analysis (Mann-Whitney) was performed using SPSS 15.0 (IBM, Nieuwegein, The Netherlands). Associations were considered statistically significant in case of a p-value ≤0.02.

## Results

### ESBL and AmpC Distribution

In the three-month study period, 1,427 ESBL screen-positive isolates were collected in the 31 participating laboratories. The first 25 isolates per laboratory, if available, comprised 723 isolates, of which 31 were excluded because of lack of viable cells or contamination with other strains, and 56 because positive screen tests could not be confirmed, leaving 636 isolates for further investigation: 479 *E. coli* (75%), 68 *E. cloacae* (11%), 67 *K. pneumoniae* (11%), 11 *K. oxytoca* (2%), and 11 *P. mirabilis* (2%). Sources and specimens of these isolates are listed in Table S1.

ESBL genes were detected in 512 of 636 isolates (81%): in 416 of 479 (87%) *E. coli,* in 64 of 67 (96%) *K. pneumoniae,* in 35 of 68 (51%) *E. cloacae*, in 6 of 11 (54%) *K. oxytoca,* and in 3 of 11 (27%) *P. mirabilis* (Table 1). Two ESBL genes were detected in 26 isolates (Table S2). Genes from the CTX-M-groups were detected most frequently (in 446 isolates), followed by SHV-genes (in 56 isolates) and TEM (in 46 isolates). Sequencing of 314 isolates revealed 16 CTX-M-variants, 3 SHV-variants, 4 TEM-variants, one GES-1 and one PER-5 (Table 2). Sequencing could not discriminate between CTX-M-15 and CTX-M-28 in 12 isolates. Nor could it discriminate between CTX-M-1 and CTX-M-61 in 2 isolates. Overall, CTX-M-15 was most prevalent, followed by CTX-M-1 and CTX-M-14 (Table 2).

**Table 1:** Identification of ESBL-groups as determined by ESBL array and PCR in 3^rd^ generation cephalosporin resistant Enterobacteriaceae.

	*E. coli*	*E. cloacae*	*K. pneumoniae*	*K. oxytoca*	*P. mirabilis*	*All species*
ESBL-group	N = 479	N = 68	N = 67	N = 11	N = 11	N = 636
CTX-M-1	301	16	48	3	2	370
CTX-M-2	2					2
CTX-M-8/25	2					2
CTX-M-9	59	11	1	1		72
SHV-2	2		4			6
SHV-4	23	14	11			48
SHV-31	1	1				2
TEM-3	28	1	2			31
TEM-4	1					1
TEM-5	2					2
TEM-17	2					2
TEM-19	10					10
GES				2		2
PER					1	1
No ESBL-gene detected	63	33	3	5	8	112

Note: 26 isolates contained 2 ESBLs.

**Table 2:** Identification of ESBL β-lactamase genes in 3^rd^ generation cephalosporin resistant Enterobacteriaceae.

ESBL-group	ESBL-gene	*E. coli*	*E. cloacae*	*K. pneumoniae*	*K. oxytoca*	*P. mirabilis*	Species
		n = 235 (75%)	n = 33 (11%)	n = 32 (10%)	n = 8 (3%)	n = 6 (2%)	n = 314
CTX-M-1	CTX-M-1	47			1		47
	CTX-M-15	80	4	20			104
	CTX-M-15/28	9		3			12
	CTX-M-22	3					3
	CTX-M-79	2					2
	CTX-M Other	2				1	3
CTX-M-9	CTX-M-9	3	3				6
	CTX-M-14	15					15
	CTX-M-17	3					3
	CTX-M-27	4					4
	CTX-M Other	4		1		1	6
	All CTX-M Variants	172	7	24	1	2	206
SHV-2	SHV-2	1		1			2
SHV-4	SHV-5			2			2
	SHV-12	13	8	3			24
	All SHV Variants	14	8	6			28
TEM-3	TEM-19	2					2
	TEM-52	13					13
TEM-5	TEM-12	1					1
TEM-19	TEM-19	1					1
	All TEM Variants	17					17
	GES-1				1		1
	PER-5					1	1
	Other ESBL Variants				1	1	2
	None of the abovegenes detected	37	19	2	5	3	66

In total 181 (28%) of 636 isolates had an AmpC phenotype; a cefoxitin MIC ≥16 mg/L (102 *E. coli* (56%), 63 *E. cloacae* (35%), 10 *K. pneumoniae* (6%), 6 *P. mirabilis* (3%)). A pAmpC gene was detected in 32 isolates: 25 of 102 (24%) *E. coli*, 3 of 10 (30%) *K. pneumoniae* and in all 4 *P. mirabilis*. These 32 isolates represented 5% of the 636 isolates (Table 3). Five different types of pAmpC β-lactamases were identified: CMY-2-group, ACT-5, MIR-1/2/3, DHA-1, and ACT-like. Sequencing did not allow for discrimination in the CMY-2 group, and MIR-1 and -2 and -3.

**Table 3:** Presence of AmpC β-lactamase genes in isolates with a MIC ≥16 mg/L for cefoxitin.

	*E. coli*	*E. cloacae*	*K. pneumoniae*	*P. mirabilis*	Species
AmpC-gene	n = 102 (56%)	n = 63 (35%)	n = 10 (6%)	n = 6 (3%)	n = 181
CMY-2 group	22		1	4	27
ACT-5	1				1
ACT-like*	1				1
MIR-1/2/3	1		1		2
DHA-1			1		1
Chromosomal	29	53**			82
None of the above genes detected	48	10	7	2	67

* GenBank Number = EF125014.1, ** = presumed.

The remaining forty-two *E. coli* isolates with AmpC-resistance phenotype and without either an ESBL or pAmpC were further investigated. Sequencing of the promoter region of the chromosomal *ampC* revealed different mutations that have been linked to resistance to third-generation cephalosporins in 29 isolates [16]–[18]. These mutations were found at the positions –1, –18,–42, –82, –88, and +58, and an insertion of an amino acid between the –10 and –35 region of the promoter. No mechanism was elucidated for the remaining 13 isolates.

### Isolate Typing

All *Klebsiella* spp., *E. coli*, and *E. cloacae* isolates were analyzed by DiversiLab (n = 625), and seven isolates appeared non-typable. For the following analysis only isolates with a detectable ESBL- or *ampC* gene were included (n = 608). When using a similarity >98% for pattern definition, 253 (53%) of 414 ESBL-producing *E. coli* had unique patterns, and cluster sizes ranged from 2 isolates (44 patterns) to 25 isolates (1 pattern) (Table 4). The overall discriminatory index of ESBL-producing *E. coli* was 0.994 (95% confidence interval (CI) 0.991–0.996). When using a similarity of >95% two large clusters emerged, one of 43 isolates (comprising 3 patterns (n = 25, n = 4, n = 4)) and one of 30 isolates (comprising 4 clusters (n = 17, n = 6, n = 5, and n = 2)). MLST typing of 21 (15 (71%) with CTX-M-15, 4 (19%) with CTX-M-1, 1 (5%) CTX-M-52, 1 (5%) with TEM-52) randomly selected isolates (11 and 10 from the cluster of 43 and 30 isolates, respectively) revealed that all belonged to ST131.

**Table 4:** Number of clusters for each cluster size in DiversiLab using >98% similarity per species.

	Diversilab cluster size												
Species (n = isolates)	1	2	3	4	5	6	7	14	17	25	not typable	Discriminatory Index	95% CI
*E. cloacae* (n = 68)	48	6	2								2	0.994	0.989–0.999
*E. coli* (n = 465)	253	44	14	2	2	1		1	1	1	2	0.994	0.991–0.996
*K. pneumoniae* (n = 64)	41	4	2				1				2	0.985	0.969–1.0
*K. oxytoca* (n = 11)	7		1								1	0.911	0.801–1.0

Forty-one (64%) of 64 *K. pneumoniae* had unique patterns, and clusters of identical patterns ranged from 2 (n = 4) to 7 (n = 1), and the discriminatory index was 0.985 (95% CI 0.969–1.0). All isolates of this cluster of 7 contained a CTX-M-1 group ESBL and sequencing of 3 of these genes revealed CTX-M-15. Forty-eight (71%) of 68 *E. cloacae* had unique patterns. There were eight clusters with 2 (n = 6) or 3 isolates (n = 2) and a discriminatory index of 0.994 (95% CI 0.989–0.999).

Inclusion of the 22 isolates without detectable ESBL or *ampC* genes did not change interpretation (data not shown).

### Association between β-lactamase Gene and Susceptibility

CTX-M-15 isolates were – on average – susceptible to 4.5 of 8 antibiotics tested, which was lower than isolates harboring TEM-52, CTX-M-1, or CTX-M-14 (mean susceptibility to 6.6, 6.0 and 5.7 antibiotics, respectively; p< = 0.004 Mann Whitney U-test) and a similar co-resistance pattern as isolates harboring SHV-12 (Table 5). There were no significant associations between β-lactamase genes and age, gender, specimen type, and institution (data now shown).

**Table 5:** Co-susceptibility according to EUCAST breakpoints in *E. coli* harboring the five most common ESBL genes.

Antibiotic	CTX-M-15 (n = 77) (%, n)	CTX-M-1(n = 44) (%, n)	SHV-12(n = 7) (%, n)	CTX-M-14(n = 15) (%, n)	TEM-52(n = 12) (%, n)
Ciprofloxacin	9 (7)	57 (25)	43 (3)	60 (9)	53 (7)
Tobramycin	21 (16)	86 (38)	43 (3)	67 (10)	100 (12)
trimethoprim/sulfamethoxazole	35 (27)	25 (11)	14 (1)	53 (8)	33 (4)
Gentamicin	56 (43)	86 (38)	57 (4)	67 (10)	92 (11)
Amikacin	60 (46)	96 (42)	74 (5)	93 (14)	100 (12)
Chloramphenicol	69 (53)	64 (28)	29 (2)	33 (5)	75 (9)
Nitrofurantoin	99 (76)	96 (42)	100 (7)	100 (15)	100 (12)
Fosfomycin	99 (76)	98 (43)	100 (7)	100 (15)	100 (12)
mean no. of co-susceptible antibiotics (range)	4.5 (2–8)	6.1 (3–8)	4.6 (3–7)	5.7 (3–8)	6.6 (5–8)

## Discussion

The population structure of third-generation cephalosporin resistant Enterobacteriaceae in the Netherlands is characterized by predominance of *E. coli* with CTX-M-15 ESBL genes, a high level of bacterial genotypic diversity, although clusters of genotypes, often *E. coli* belonging to MLST131, were observed in individual laboratories. AmpC type resistance was observed in 53 *E. coli* isolates (11% of all *E.* coli) and resulted from pAmpC genes, mostly being *bla*_CMY-2_, or *ampC* promoter mutations in equal frequencies.

The predominance of the CTX-M-gene family among the ESBL genes in Dutch isolates has also been observed in Belgium, France, Italy, Poland, Spain and Canada. [6]–[8], [20]–[22] As in the Netherlands, CTX-M-15 was most prevalent within the CTX-M family in all these countries, except in Poland and Spain were CTX-M-3 and CTX-M-14 were more prevalent. [7], [21].

*E. coli* ST131 carrying IncFII plasmids with CTX-M-15 is considered the most important disseminator of CTX-M-15 worldwide. Our finding of clusters of ST131 isolated within a short time-frame in single laboratories suggests the occurrence of clonal dissemination of ESBL-producing ST131 *E. coli*. In our study most of the ST 131 *E. coli* contained CTX-M-15, and presence of this gene was associated with – on average – higher levels of resistance. Whether the clonal spread of ST131 occurs in health care-facilities or in the community remains to be determined as in the present study ST131 was in equal numbers obtained from samples submitted from health care institutes or by GPs. As such we agree with current literature that the origin ST131 remains unclear. [23].

The high prevalence of CTX-M-1 and TEM-52 in *E. coli* in the Netherlands may result from food-borne exposure, as poultry and retail meat are frequently contaminated with *E. coli* harbouring these genes on identical plasmids as found in human isolates. [24] Similar findings (albeit with lower prevalence of TEM-52) have been reported from Belgium, where retail meat was also frequently contaminated with ESBL-producing *E. coli* and which country shares food distributors with the Netherlands, and north Italy. [20], [22] In contrast, TEM-19 rather than TEM-52 is the most prevalent TEM β-lactamase in Spain and Poland. [7], [21].

Five percent of *E. coli* and 4% of *K. pneumoniae* conferring resistance to third generation cephalosporins carried pAmpC beta-lactamases. Little is known about the epidemiology of CMY-group beta-lactamases. In Poland CMY-12 and CMY-15 were predominant in *E. coli* and in the UK and Ireland pAmpCs of the CIT group, which includes the CMY-2 group, and genes belonging to the FOX and ACC family were detected in *E. coli* and *K. pneumoniae* [7], [25]. The source of CMY-2 is unknown. Although CMY-2 genes have been identified in poultry and poultry meat in Belgium, Spain and the Netherlands [26]–[28], more detailed studies are needed to demonstrate the relevance and frequency of gene or strain transmission between both reservoirs.

All *E. coli*, except three isolates, resistant to cefoxitin in which no pAmpC beta-lactamases or mutated promoters were detected, contained ESBL genes, which may explain their resistance phenotype. In the other three isolates the increased MIC for cefoxitin may have resulted from mechanisms not investigated in this study, e.g. porin mutations, alteration in the expression of efflux pumps and/or porins, and mutations in the target of β-lactamases.

In this study, derepressed chromosomal *ampC* genes and pAmpC beta-lacmtase genes were equally prevalent in *E. coli.* This has also been observed in a French study [29], but a higher prevalence of derepressed chromosomal *ampC* genes was detected in Belgium. [30] This difference could result from differences in selection of isolates.

In 636 isolates with phenotypic resistance to third-generation cephalosporins 551 ESBLs and 82 AmpCs (including 53 assumed chromosomal *ampC* genes in *E.* cloacae) were detected in 610 (96%) isolates. In the remaining 26 isolates (14 *E. coli*, 5 *K. oxytoca*, 4 *P. mirabilis*, and 3 *K. pneumoniae*) resistance may have been caused by mechanisms not investigated in this study, such as the presence of OXA genes, hyperproduction of chromosomal OXY genes in *K. oyxtoca*, porin mutations, alteration in the expression of efflux pumps and/or porins, and mutations in the target of the β-lactamases.

Although we consider the selected isolates to be representative for the Netherlands, there may have been some selection bias as we did not adjust isolate selection on the size of the catchment populations of the different laboratories.

Our findings demonstrate that in the Netherlands *bla*_CTX-M-15_ is the most prevalent cause of third-generation cephalosporin resistance in the Netherlands and that resistance due to either hyperproduction of chromosomal *ampC* or plasmid-borne AmpC beta-lactamases occurs more frequently than previously reported.

## Supporting Information

Table S1 Material and provider of the isolates grouped by species.
(DOC)

Table S2 Combinations of β-lactamases expressed in one isolate in the primary selection and random sample.
(DOC)
