# Correction: Population Distribution of Beta-Lactamase Conferring Resistance to Third-Generation Cephalosporins in Human Clinical Enterobacteriaceae in The Netherlands

**DOI:** 10.1371/journal.pone.0107786

**Published:** 2014-09-08

**Authors:** The PLOS ONE Staff

The formatting and spelling of the last Author’s name is incorrect. The correct name is: Maurine A. Leverstein-van Hall.
