# Supplementary material for: Population Distribution of Beta-Lactamase Conferring Resistance to Third-Generation Cephalosporins in Human Clinical Enterobacteriaceae in The Netherlands
Source: PLoS One. 2012 Dec 20;7(12):e52102. doi: 10.1371/journal.pone.0052102 (PMC3527366; doi:10.1371/journal.pone.0052102)
Supplement: Table S1 — Material and provider of the isolates grouped by species. (DOC) [file pone.0052102.s001.doc]

|  | *E. cloacae*  (n=68) | *E. coli*  (n=479) | *K. oxytoca*  (n=11) | *K. pneumoniae*  (n=67) | *P. mirabilis*  (n=11) |
| --- | --- | --- | --- | --- | --- |
| **Material** |  |  |  |  |  |
| Ascites | 3 | 5 |  | 1 |  |
| Blood | 1 | 10 |  | 2 |  |
| Gynaecological |  | 3 |  | 15 |  |
| Pulmonary | 8 | 29 | 5 |  | 2 |
| Rectum/faeces | 12 | 58 | 3 | 18 |  |
| Urine | 33 | 333 | 2 | 25 | 7 |
| Wound/Abscess/Skin | 11 | 39 | 1 | 5 | 2 |
| Other/Unknown |  | 2 |  | 1 |  |
| **Provider** |  |  |  |  |  |
| Academic Hospital | 1 | 23 | 2 | 14 | 1 |
| Non-Academic Hospital | 52 | 259 | 8 | 35 | 7 |
| Long-term Care Facility | 3 | 26 |  | 4 | 2 |
| General Practitioner | 10 | 147 |  | 12 | 1 |
| Other/Unknown | 2 | 24 | 1 | 2 |  |
