# Supplementary material for: Population Distribution of Beta-Lactamase Conferring Resistance to Third-Generation Cephalosporins in Human Clinical Enterobacteriaceae in The Netherlands
Source: PLoS One. 2012 Dec 20;7(12):e52102. doi: 10.1371/journal.pone.0052102 (PMC3527366; doi:10.1371/journal.pone.0052102)
Supplement: Table S2 — Combinations of β-lactamases expressed in one isolate in the primary selection and random sample. (DOC) [file pone.0052102.s002.doc]

| **ESBL-ESBL combinations** | **Species** | **n** | **β-lactamase genotypes** |
| --- | --- | --- | --- |
| CTX-M-1 gr. & CTX-M-9 gr. | *E. cloacae* | 2 | Not Typed (n=2) |
|  | *E. coli* | 1 | CTX-M-15 & CTX-M-27 (n=1) |
| CTX-M-1 gr. & CTX-M-8/25 gr. | *E. coli* | 1 | CTX-M-15 (CTX-M-8/25-gr. Not Typed) (n=1) |
| CTX-M-1 gr. & SHV-4 gr. | *E. cloacae* | 2 | CTX-M-15 & SHV-12 (n=1) |
|  |  |  | Not typed (n=1) |
|  | *E. coli* | 6 | CTX-M-1/61 & SHV-12 (n=2) |
|  |  |  | CTXM-15 & SHV-12 (n=2) |
|  |  |  | Not typed (n=2) |
| CTX-M-9 gr. & SHV-4 gr. | *E. cloacae* | 3 | CTX-M-9 & SHV-12 (n=1) |
|  |  |  | SHV-12 (CTX-M-9-gr. not typed) (n=1) |
|  |  |  | Not typed (n=1) |
|  | *E. coli* | 1 | CTX-M-9 & SHV-12 (n=1) |
|  | *K. pneumoniae* | 1 | Not typed (n=1) |
| CTX-M-1 gr. & TEM-3 gr. | *E. coli* | 2 | CTX-M-1/61 & TEM-52 (n=1) |
|  |  |  | Not typed (n=1) |
| CTX-M-1 gr. & TEM-19 gr. | *E. coli* | 2 | CTX-M-15 (TEM-19-gr. not typed) (n=1) |
|  |  |  | TEM-19 (CTX-M-1-gr. not typed) (n=1) |
| CTX-M-1 gr. & TEM-4 gr. | *E. coli* | 1 | Not typed (n=1) |
| SHV-2 gr. & TEM-19 gr. | *E. coli* | 1 | Not typed (n=1) |
| SHV-31 gr. & TEM-3 gr. | *E. cloacae* | 1 | Not typed (n=1) |
|  | *E. coli* | 1 | Not typed (n=1) |
| SHV-4 gr. & TEM-25 gr. | *E. coli* | 1 | SHV-12 & TEM-25 (n=1) |
|  |  |  |  |
| **ESBL-AmpC combinaties** | **Species** | **n** | **ESBL Genotypes** |
| CTX-M-1 gr. & CIT | *E. coli* | 4 | CTX-M-1 (CMY not typed) (n=1) |
|  |  |  | CTX-M-15 (CMY not typed) (n=2) |
|  |  |  | Not typed (n=1) |
|  | *K. pneumoniae* | 1 | Not typed (n=1) |
| CTX-M-1 gr. & MIR | *E. coli* | 1 | CTX-M-15 & MIR-1/2/3 (n=1) |
| SHV-4 gr. & MIR | *K. pneumoniae* | 1 | MIR-1/2/3 (SHV-4-gr. not typed) (n=1) |
|  |  |  |  |
| **ESBL-ESBL-AmpC combinaties** | **Species** | **n** | **ESBL Genotypes** |
| CTX-M-1 gr. & SHV-4 gr. & DHA | *K. pneumoniae* | 1 | DHA-1 |
